# Associations Between Simulated Future Changes in Climate, Air Quality, and Human Health

**DOI:** 10.1001/jamanetworkopen.2020.32064

**Published:** 2021-01-04

**Authors:** Neal L. Fann, Christopher G. Nolte, Marcus C. Sarofim, Jeremy Martinich, Nicholas J. Nassikas

**Affiliations:** 1 Office of Air Quality Planning and Standards, Office of Air and Radiation, US Environmental Protection Agency, Research Triangle Park, North Carolina; 2 Center for Environmental Measurement and Modeling, Office of Research and Development, US Environmental Protection Agency, Research Triangle Park, North Carolina; 3 Office of Atmospheric Programs, Office of Air and Radiation, US Environmental Protection Agency, Washington District of Columbia; 4 Department of Pulmonary, Critical Care, and Sleep Medicine, Alpert School of Medicine, Brown University, Providence, Rhode Island

## Abstract

**Importance:**

Future changes in climate are likely to adversely affect human health by affecting concentrations of particulate matter sized less than 2.5 μm (PM_2.5_) and ozone (O_3_) in many areas. However, the degree to which these outcomes may be mitigated by reducing air pollutant emissions is not well understood.

**Objective:**

To model the associations between future changes in climate, air quality, and human health for 2 climate models and under 2 air pollutant emission scenarios.

**Design, Setting, and Participants:**

This modeling study simulated meteorological conditions over the coterminous continental US during a 1995 to 2005 baseline and over the 21st century (2025-2100) by dynamically downscaling representations of a high warming scenario from the Community Earth System Model (CESM) and the Coupled Model version 3 (CM3) global climate models. Using a chemical transport model, PM_2.5_ and O_3_ concentrations were simulated under a 2011 air pollutant emission data set and a 2040 projection. The changes in PM_2.5_ and O_3_-attributable deaths associated with climate change among the US census-projected population were estimated for 2030, 2050, 2075, and 2095 for each of 2 emission inventories and climate models. Data were analyzed from June 2018 to June 2020.

**Main Outcomes and Measures:**

The main outcomes were simulated change in summer season means of the maximum daily 8-hour mean O_3_, annual mean PM_2.5_, population-weighted exposure, and the number of avoided or incurred deaths associated with these pollutants. Results are reported for 2030, 2050, 2075, and 2095, compared with 2000, for 2 climate models and 2 air pollutant emissions data sets.

**Results:**

The projected increased maximum daily temperatures through 2095 were up to 7.6 °C for the CESM model and 11.8 °C for the CM3 model. Under each climate model scenario by 2095, compared with 2000, an estimated additional 21 000 (95% CI, 14 000-28 000) PM_2.5_-attributable deaths and 4100 (95% CI, 2200-6000) O_3_-attributable deaths were projected to occur. These projections decreased to an estimated 15 000 (95% CI, 10 000-20 000) PM_2.5_-attributable deaths and 640 (95% CI, 340-940) O_3_-attributable deaths when simulated using a future emission inventory that accounted for reduced anthropogenic emissions.

**Conclusions and Relevance:**

These findings suggest that reducing future air pollutant emissions could also reduce the climate-driven increase in deaths associated with air pollution by hundreds to thousands.

## Key Points

**Question:**

What is the association of reducing air pollutant emissions with the human health burden associated with climate change?

**Findings:**

In this modeling study, projected changes in climate and concentrations of ozone (O_3_) and particulate matter smaller than 2.5 μm in diameter (PM_2.5_) were estimated over the 21st century under a high–greenhouse gas scenario using 2 climate models and 2 air pollutant emission data sets. The substantial increases in estimated air pollution–attributable mortality associated with climate change were projected to decline if air pollutant emissions were reduced.

**Meaning:**

These findings suggest that reducing air pollutant emissions could attenuate but not eliminate the climate change-induced increase in mortality associated with air pollution.

## Teaser

This modeling study uses 2 climate models and under 2 air pollutant emission scenarios to model the associations between future changes in climate, air quality, and human health.

## Introduction

Future changes in the climate will affect the level and distribution of common air pollutants, including ground-level ozone (O_3_) and fine particles sized 2.5 μm and smaller (PM_2.5_) in the United States.^1,2,3,4,5^ The climate can affect pollutant concentrations through 2 broad pathways. In the first, changes in the climate can alter meteorological variables, including temperature, cloud cover, humidity, precipitation, and wind patterns—each of which influence the production of O_3_ and PM_2.5._^6,7,8^ These climate-related changes in meteorological conditions also influence emissions of volatile organic compounds (VOCs) as well as naturally occurring events, including wildland fires and windblown dust, which each emit PM_2.5_.^9^

The human health risks associated with these pollutants are well established in controlled human exposure studies and toxicology and epidemiology literature.^10,11,12^ Myocardial infarctions, strokes, and respiratory diseases, such as chronic obstructive lung disease, acute lower respiratory tract infections, and lung cancer, are the primary drivers of mortality associated with air pollution.^12,13^ The mechanisms of these deaths are likely due to oxidative stress, alterations in host immune defense, increased permeability of endothelial cells, and localized and systemic inflammation.^10,14^ Applying a suite of tools to model the pathways from climate to air quality and ultimately human health, researchers have quantified estimated counts of air pollution-related premature death and illness attributable to future climate change.^15,16^ Often, these analyses run a global or regional climate model for 1 or more climate scenarios, projecting climate-induced changes in meteorological variables.^17^ Downscaled meteorological projections are input to photochemical air quality models, which simulate the concentrations of air pollutants. Finally, these model-projected changes in pollutants are input to a human health impact assessment.

The number and economic value of these estimated outcomes can be substantial. For example, the 2017 US Environmental Protection Agency Climate Change Impacts and Risk Analysis project estimated O_3_-attributable premature deaths associated with future changes in climate using the Community Climate System Model (version 4) under 2 Representative Concentration Pathway (RCP) scenarios: RCP8.5 (simulating 8.5 W/m^2^ radiative forcing), a high–greenhouse gas emissions scenario, and RCP4.5 (simulating 4.5 W/m^2^ radiative forcing), a climate stabilization scenario.^18,19,20^ That analysis applied a global climate model, a chemical transport model, and a health impacts assessment tool to quantify climate, air quality, and health impacts. The Climate Change Impacts and Risk Analysis project estimated 420 to 1200 premature deaths in 2050 and 920 to 2500 premature deaths in 2090 associated with climate-related changes in O_3_ under the RCP8.5 scenario, as well as morbidity outcomes, including hospital and emergency department visits.^21^ That study valued these impacts at $9.8 billion in 2050 and $26 billion in 2090.

Similar studies over the past decade have aimed to define the scope and magnitude of the health burden associated with climate-induced changes in air quality so as to better quantify the climate penalty,^15,22,23,24^ defined as the excess risks to human health associated with climate-induced changes in air quality. Researchers have assessed climate-related air pollution health outcomes associated with PM_2.5_ and O_3_,^25,26^ early-century climate change,^16^; alternative climate models,^16^ and outcomes simulated using climate models informed by the Intergovernmental Panel on Climate Change Fourth Assessment Report emission scenarios.^27,28^ To our knowledge, this is the first study to estimate mortality associated with both near-term and longer-term changes in PM_2.5_ and O_3_ using 2 climate models and 2 air pollutant emissions scenarios.

This study builds on the literature by addressing 2 questions: what are the estimated climate-related impacts over the 21st century under a high-warming scenario in the form of deaths associated with O_3_ and PM_2.5_ and to what extent will reducing anthropogenic emissions mitigate these estimated impacts.

## Methods

This study was exempt from institutional review board approval because it did not include human participants, per US Environmental Protection Agency policy. This study is reported following the Consolidated Health Economic Evaluation Reporting Standards (CHEERS) reporting guideline.

### Climate Modeling

This analysis uses modeling of RCP8.5, a scenario in which accumulated greenhouse gas concentrations lead to 8.5 W/m^2^ of radiative forcing (ie, warming) (eAppendix in the Supplement) in the year 2100.^18^ Simulations from 2 global climate models,—the Community Earth System Model (CESM) and the Geophysical Fluid Dynamics Laboratory Coupled Model version 3 (CM3)—were dynamically downscaled to 36-km resolution over North America using the Weather Research and Forecasting model.^29^ Owing to computational constraints, only 1 greenhouse gas scenario was modeled. RCP8.5 was selected to assess a wide range of future climates, but this does not imply a judgment regarding the likelihood of that scenario: recent research, such as Christensen et al,^30^ suggests that even in the absence of any global climate policy, RCP8.5 has a higher forcing than the most likely future concentration pathway.

### Air Quality Modeling

Applying these downscaled meteorological conditions, the Community Multiscale Air Quality (CMAQ) simulated air quality over the coterminous US for five 11-year periods in the 21st century, centered on 2000, 2030, 2050, 2075, and 2095.^31^ For each climate model and year, CMAQ was run using 2 anthropogenic air pollutant emissions data sets based on the 2011 National Emissions Inventory: a base case using the 2011 emissions inventory and a year 2040 projection.^32^ The National Emissions Inventory estimates the level and distribution of pollutants emitted from all sources, while the 2040 emissions inventory projection accounts for the implementation of a suite of federal, state, and local air quality policies on stationary and mobile sources. Controlled stationary emission sources include electricity-generating units, industrial boilers, cement kilns, pulp and paper facilities, and other sources that would reduce precursor emissions and potentially influence the climate penalty. Controlled mobile sources include on-road vehicles, marine vessels, and locomotives. Policies controlling emissions from these sectors are projected to reduce PM_2.5_ and O_3_ precursor emissions substantially, with nitrogen oxides decreasing 44%, sulfur dioxide (SO_2_) decreasing 57%, and VOC emissions decreasing 12% between 2011 and 2040 (eTable 1 in the Supplement). Vegetative emissions of VOCs were simulated using the downscaled meteorological conditions, and thus respond to the changes in climate. The effects of meteorological conditions on other emissions processes are neglected, including changes in evaporative emissions of VOCs from liquid fuels and solvents as well as changes in PM_2.5_ emissions from wildfires and dust storms.

### Quantifying Population-Weighted Exposure and Premature Deaths Associated with O_3_ and PM_2.5_

The number of premature deaths associated with O_3_ and PM_2.5_ exposure were estimated using an approach documented elsewhere in the literature.^33,34,35^ The open-source environmental Benefits Mapping and Analysis Program–Community Edition (BenMAP-CE) software program quantified PM_2.5_- and O_3_-attributable premature deaths in each of the 4 future periods compared with the 2000 baseline.^36^ The BenMAP-CE program calculates health impacts using a concentration-response parameter between O_3_ or PM_2.5_ and the risk of premature death, the population exposed to O_3_ or PM_2.5_, the baseline death rate of that population, and the change in O_3_ or PM_2.5_ the population experiences.

To calculate estimated PM_2.5_-attributable premature deaths, counts of total deaths associated with PM_2.5_ (y_ij_) were calculated during each period *i* (*i* = 2000, 2030, 2050, 2075, and 2095) among adults aged 30 years and older (*a*) in each county *j* (j = 1,…,J in which J is the total number of counties) as

*y_ij_* = Σ*_a_ y_ija_*

*y_ija_* = *mo_ija_* × (*e*^β∙^*^Cij^*-1) × *P_ija_*

in which *mo_ija_* is the baseline all-cause mortality rate for adults aged 30 to 99 years (*a*) in county *j* in year *i* stratified in 10-year age groups, β is the risk coefficient for all-cause mortality for adults associated with PM_2.5_ exposure, *C_ij_* is annual mean PM_2.5_ concentration in county *j* in year *i*, and *P_ija_* is the number of adult residents aged 30 to 99 years in county *j* in year *i* stratified into 5-year age groups. When calculating outcomes, the program assigns the 10-year stratified death rate to the corresponding 5-year stratified population bin. The health outcome function used to calculate deaths associated with daily changes in O_3_ is the same, with the exception of the baseline death rate, which is expressed as a daily, rather than an annual, rate. The program performs a Monte Carlo analysis by randomly sampling from a distribution constructed from the SE reported for each study; the resulting distribution is then used to report 95% CIs.

#### Selecting Concentration-Response Parameters

The extended analysis of the American Cancer Society cohort by Krewski et al^37^ provides the parameter for a concentration-response association quantifying new cases of PM_2.5_-attributable premature deaths. This risk coefficient has been broadly applied in the literature and expresses the association between long-term PM_2.5_ exposure and all-cause death associated with PM_2.5_ (hazard ratio 1.06 [95% CI, 1.04-1.08] per 10 μg/m^3^ increase in mean PM_2.5_ concentrations in 1999-2000, adjusted for all individual-level and ecological covariates). This function applies to the US adult population aged 30 years and older.

O_3_-attributable premature deaths were quantified using a concentration-response parameter from a 2008 multicity time series study of 48 cities.^38^ That study by Zanobetti and Schwartz^38^ reported an effect size of 0.53% (95% CI, 0.28%-0.77%) per 10 ppb in maximum daily 8 hours O_3_ for lag 0 to 3 days all-cause mortality among populations of all ages (eTable 2 in the Supplement). The US Environmental Protection Agency recently published an Integrated Science Assessment for Ozone^10^ in which it indicated that the evidence is “suggestive of, but not sufficient to infer, a causal relationship” with total mortality not caused by unintentional injury.^33^ However, the evidence remains supportive of a causal relationship with short-term respiratory effects, including mortality, and analyses published elsewhere that the estimated number of O_3_-attributable respiratory premature deaths is approximately commensurate with the estimated number of deaths not caused by unintentional injury.^34,35^

#### Demographic and Baseline Health Parameters

The BenMAP-CE tool contains projected cause-specific and age-stratified crude death rates through the year 2060 at the US county level. A 3-year mean of the baseline rates of all-cause mortality from the US Centers for Disease Control and Prevention WONDER database for the years 2012 to 2014 were calculated and then projected to future years by drawing on US Census Bureau projected mortality rates, available in 5-year increments through the year 2060.^39,40,41^ Outcomes in the years 2075 and 2095 were calculated using the 2060 death rates (eTable 3 and eTable 4 in the Supplement).

The Integrated Climate and Land Use Scenarios (version 2) supplied county-level age-stratified projected population counts to the year 2095.^42^ The Integrated Climate and Land Use Scenarios were harmonized with the median variant projection of the United Nations’ 2015 World Population Prospects data set, a midrange population projection similar to Shared Socioeconomic Pathway 2.^43,44^ Data were analyzed from June 2018 to June 2020.

## Results

### Climate and Air Quality Modeling Results

The simulated downscaled meteorological projections increased annual mean temperatures through late century, increasing from 2.0 °C to 6.6 °C between 2030 and 2095 under CM3 and 1.5 °C to 4.7 °C under CESM (eTable 5 in the Supplement). By 2095, CESM projected daily maximum temperatures during summer to increase by a mean of 5.1 °C across the continental US, while CM3 projected maximum temperatures to increase by 7.6 °C, each relative to a 2000 baseline (eFigure 1 and eFigure 2 in the Supplement); these changes are consistent with those reported elsewhere in the literature.^45^ CESM projected the highest temperature increases in the midwestern US, while CM3 predicted the highest temperature increases in the intermountain West.

Under each climate scenario and air pollutant emissions data set, the summer mean of the maximum daily 8-hour O_3_ concentration increased over parts of the US (≤13 ppb) while decreasing in others compared with the early century (≤6 ppb) (eFigure 3 in the Supplement). The spatial distribution of these changes was generally consistent within a particular climate model for each future year, but not between models. For example, meteorological conditions downscaled from CESM led to greater mean maximum daily 8-hour O_3_ concentration increases in the upper midwestern, eastern, and southwestern US (≤11 ppb). By contrast, driving CMAQ with meteorological conditions downscaled from CM3 led to the greatest mean maximum daily 8-hour O_3_ concentration increases in the Great Plains and midwestern US (≤13 ppb). Both climate models projected decreases in O_3_ concentrations in the southern US. CESM and CM3 each projected increased annual mean PM_2.5_ concentrations in the southeastern US (≤4.3 μg/m^3^) and decreasing PM_2.5_ levels in the midwestern US (≤1.5 μg/m^3^).

### Estimated Air Pollution Exposure and Health Impacts

Future changes in climate will likely alter the level and distribution of annual mean PM_2.5_ concentrations and summer season O_3_ concentrations, which would increase population exposure to these 2 pollutants compared with the year 2000 baseline by as much as 3.6 ppb for O_3_ and 0.65 μg/m^3^ for PM_2.5_ (eTable 6 in the Supplement). The estimated climate-driven population-weighted exposure to PM_2.5_ was projected to increase with increasing temperatures; this is consistent across climate models and emissions inventories. By contrast, the CM3-simulated population-weighted exposure to summer season O_3_ was projected to decrease in 2030 and 2050 compared with 2000 under the projected 2040 emissions inventory (a result of reducing O_3_ precursor emissions under this inventory) but increase for all other scenarios modeled. For each model and for each year, the modeled 2040 emission inventory yielded lower levels of estimated PM_2.5_ and O_3_ population exposure than the 2011 inventory.

Our models found that reducing anthropogenic emissions through the year 2040 was associated with thousands of fewer estimated PM_2.5_- and O_3_-attributable premature deaths per year (Table). Using the 2011 emissions inventory and compared with 2000, by 2095, an estimated additional 21 000 (95% CI, 14 000-28 000) PM_2.5_-attributable deaths and 4100 (95% CI, 2200-6000) O_3_-attributable deaths were projected to occur. When simulated using a future emission inventory that accounted for reduced anthropogenic emissions, an estimated additional 15 000 (95% CI, 10 000-20 000) PM_2.5_-attributable deaths and 640 (95% CI, 340-940) O_3_-attributable deaths were projected to occur, compared with 2000. The relative number of avoidable premature deaths associated with climate-driven changes in PM_2.5_ and O_3_ varied by climate model and inventory. For example, for many of the CESM scenarios, we estimated a greater number of avoidable deaths attributable to O_3_ than to PM_2.5_ (as many as 2100 [95% CI, 1100 to 3100] O_3_-attributable deaths vs 290 [95% CI, 180 to 360] PM_2.5_-attributable deaths), but under the CM3 scenarios, the estimated number of PM_2.5_-attributable avoidable deaths was more similar to the estimated number of O_3_-attributable avoidable deaths (as many as 6200 [95% CI, [4200 to 8300] PM_2.5_-attributable deaths vs 3500 [95% CI, 1900 to 5100] O_3_-attributable deaths).

**Table.** Estimated Number of Avoided O_3_- and PM_2.5_-Attributable Premature Deaths Associated With Controlling Future Levels of Anthropogenic Emissions^a^

Year	Deaths, No. (95% CI)			
	CM3		CESM	
	PM_2.5_	O_3_	PM_2.5_	O_3_
2030	1600 (1000 to 2100)	650 (350 to 950)	290 (200 to 390)	480 (260 to 710)
2050	2500 (1700 to 3400)	1400 (740 to 2000)	−630 (−430 to −830)	720 (390 to 1100)
2075	5600 (3800 to 7400)	2500 (1300 to 3600)	45 (29 to 63)	1400 (740 to 2000)
2095	6200 (4200 to 8300)	3500 (1900 to 5100)	270 (180 to 360)	2100 (1100 to 3100)

Abbreviations: CESM, Community Earth System Model; CM3, Coupled Model version 3; O_3_, ozone; PM_2.5_, particulate matter sized 2.5 μm and smaller.

^a^ Projections are based on a 2040 emission inventory compared with a 2011 emission inventory (Representative Concentration Pathway 8.5 scenario).

The mortality burden associated with PM_2.5_- and O_3_ was substantial and not distributed equally across the US (eTable 7 in the Supplement). Projected PM_2.5_ and O_3_ concentrations, combined with heterogeneous population density and baseline death rates, each influence the estimated number of deaths avoided or incurred. The southeastern US was projected to experience the greatest number of PM_2.5_- and O_3_-attributable deaths incurred or avoided for each climate model and inventory by late century (as many as 11 000 PM_2.5_-attributable deaths and 580 O_3_-attributable deaths in 2095). The northwestern US was projected to experience a comparatively smaller number of deaths (as few as 260 PM_2.5_-attributable deaths and 48 O_3_-attributable deaths). The Midwest was projected to experience a substantial number of avoided PM_2.5_-attributable deaths, irrespective of year, emissions inventory, or climate model (as many as 2800 avoided deaths).

**Figure 1.**
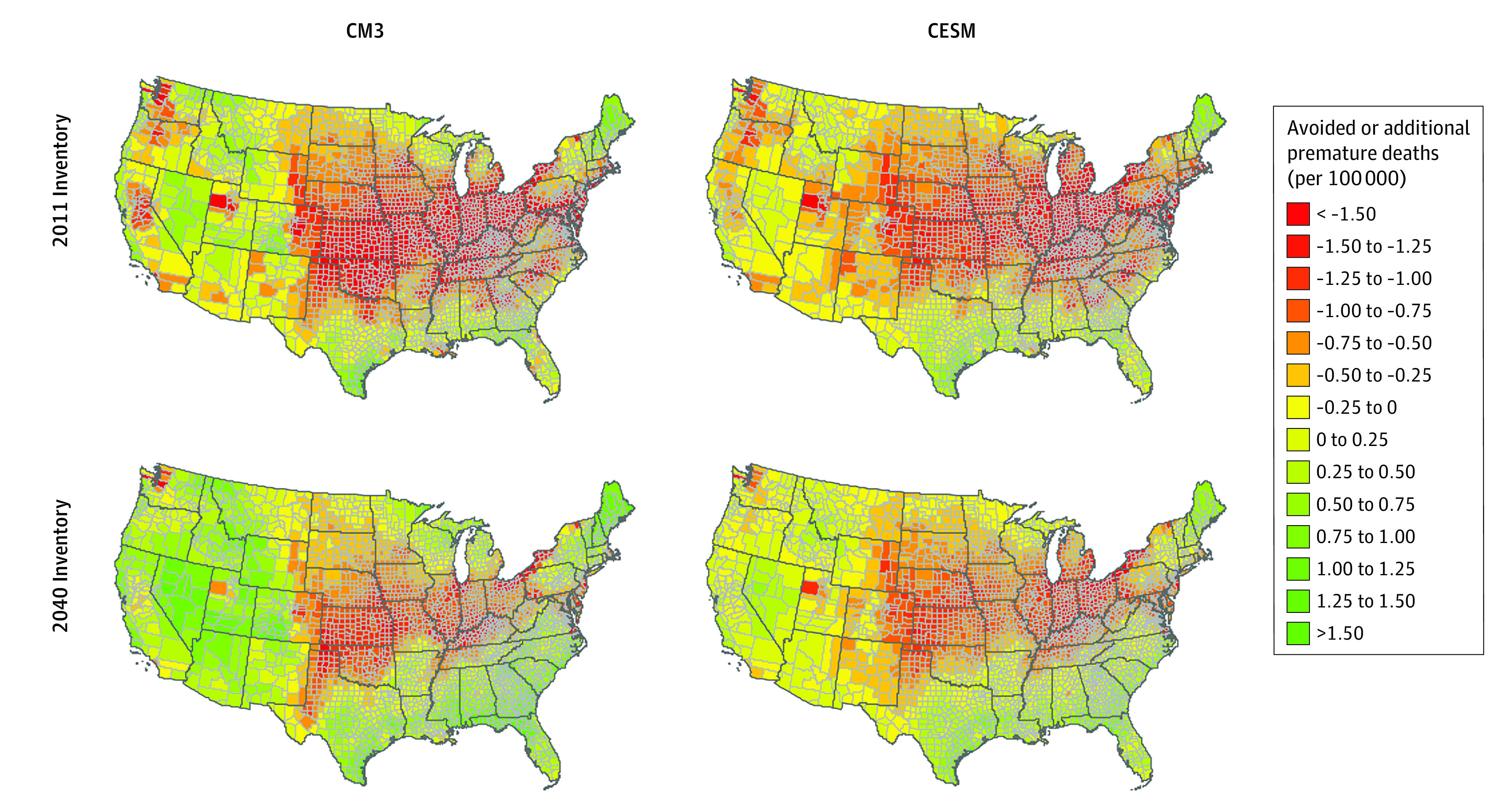
Estimated Premature Deaths Associated With Ozone Concentrations in 2095 per 100 000 Population Estimated using 2011 and 2040 emissions inventories from Coupled Model version 3 (CM3) and Community Earth System Model (CESM) compared with a base year of 2000, Representative Concentration Pathway 8.5 scenario.

**Figure 2.**
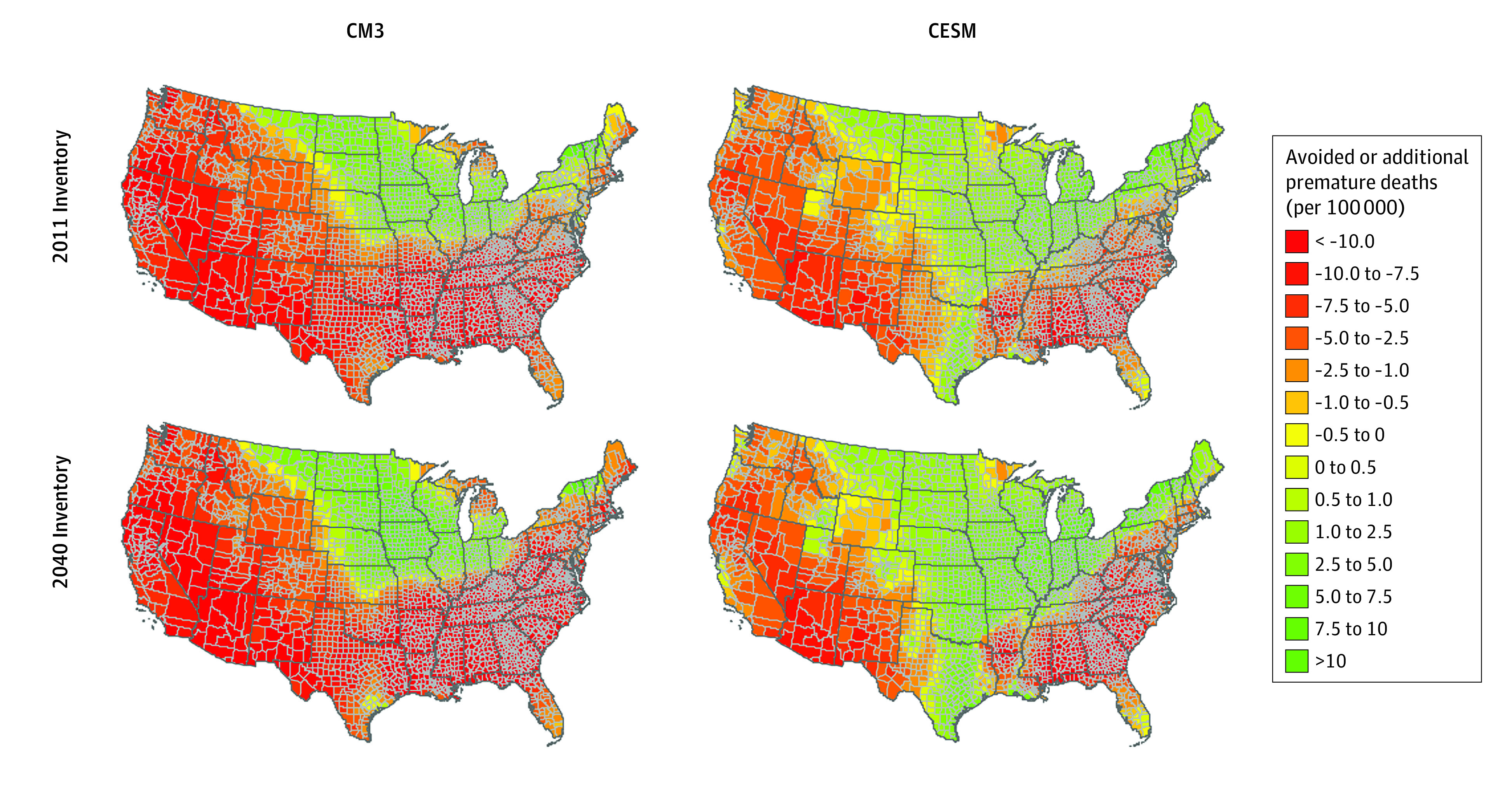
Estimated Premature Deaths Associated With Concentrations of Particulate Matter 2.5 μm and Smaller in 2095 per 100 000 Population Estimated using 2011 and 2040 emissions inventories from Coupled Model version 3 (CM3) and Community Earth System Model (CESM) compared with a base year of 2000, Representative Concentration Pathway 8.5 scenario.

The estimated PM_2.5_- and O_3_-attributable deaths occurring in late century were substantial and projected to affect most of the continental US (Figure 1 and Figure 2). Estimated O_3_-attributable death rates (calculated as events per 100 000 people in each county) were projected to be greatest among counties in the Midwest and Great Plains and to a lesser extent the Northwest (as high as 1.5 deaths per 100 000) (Figure 1). These results are consistent across the 2 climate models. The projected O_3_-attributable premature deaths were attenuated significantly when using a 2040 emissions inventory, particularly among counties in the Northwest and to a lesser extent the Southeast. The late-century projected mortality associated with PM_2.5_ burden was greatest in the western US, southern Great Plains and southeastern US (as high as 10 deaths per 100 000) (Figure 2). Using a 2040 emissions inventory attenuated the late-century mortality burden associated with PM_2.5_ compared with using a 2011 emissions inventory. Implementing the 2040 emission inventory was projected to reduce the overall mortality burden associated with PM_2.5_ and O_3_ compared with the 2011 inventory (eTable 7 in the Supplement).

### Association of PM_2.5_- and O_3_-Attributable Deaths With Annual Temperature

**Figure 3.**
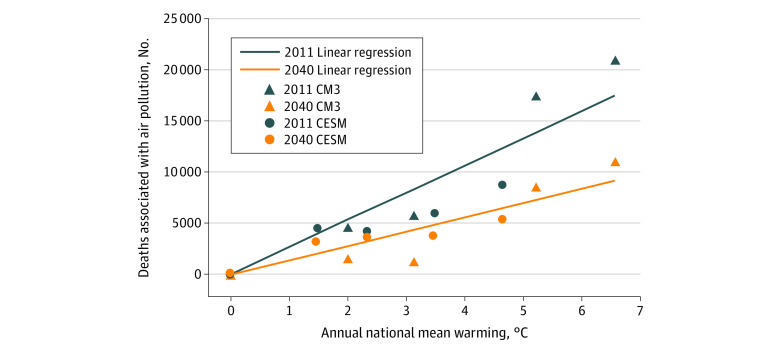
Estimated Deaths Associated With Particulate Matter Sized 2.5 μm and Smaller and Ozone Concentrations by Temperature Estimated using 2011 and 2040 emissions inventories from Coupled Model version 3 (CM3) and Community Earth System Model (CESM) with Linear Fits.

The estimated counts of deaths associated with air pollution were associated with annual temperature in each year (Figure 3); this analysis gives insight to the association between temperature and the air pollution mortality burden in each year. To control for the influence of population growth, population counts were fixed to the year 2025 in each scenario. A linear regression estimated a climate mortality penalty of approximately 2700 (SE, 250) deaths associated with air pollution per degree of mean national warming for the 2011 inventory compared with 1400 (SE, 140) deaths per degree for the 2040 inventory (Figure 3).

## Discussion

In this modeling study, we estimated hundreds to thousands of PM_2.5_- and O_3_-attributable premature deaths per year due to climate change. The direction and magnitude of these outcomes were generally consistent among 2 climate models, although they varied by US region. We also observed that reducing air pollutant emissions—in the form of directly-emitted PM_2.5_ and PM_2.5_ precursor emissions, such as nitrogen oxides and SO_2_—was projected to mitigate the future impact of climate change on air quality and health. The 2040 emissions inventory, which assumed the institution of policies to reduce precursor emissions, projected a smaller climate-driven increase in population-weighted PM_2.5_ and summer season O_3_ compared with the 2011 emissions inventory for both climate models. This suggests that reducing emissions would not only directly reduce population exposure to those pollutants, but also reduce the climate penalty, yielding additional benefits to reducing precursor emissions.

Future changes in the climate will alter meteorological variables, which would, in turn, likely affect the level and distribution of air pollutants, including O_3_ and PM_2.5_.^46,47^ While this fact is well established in the literature, the direction and magnitude of the change in these pollutants (and hence the effect on public health) are less clear. For example, some studies project decreased climate-attributable O_3_ concentrations and associated health outcomes in the early century in certain regions,^16,25^ while other models project substantial increases in O_3_ concentrations and associated health outcomes by midcentury.^28,48^ Other literature, such as a 2017 study by Garcia-Menendez et al,^49^ suggests that natural variability contributes to uncertainty in projected O_3_ concentrations.

Comparing mortality associated with air pollution with annual national mean temperature change provides a reduced-form technique for quantifying excess deaths associated with air pollution. As future changes in temperature can be calculated readily from reduced complexity models, this approach allows mortality associated with climate change–driven air pollution to be estimated for other scenarios or time periods. However, our analysis considered only 2 climate models under 2 levels of air pollutant emissions for 4 temperature increments, providing limited data with which to determine whether a nonlinear function might better describe the association between national temperatures and mortality associated with air pollution. Including more models in the future would enable us to better characterize the association between temperature and mortality.

### Limitations

This study has some limitations. Modeling climate-induced change in fine particle levels is made especially challenging by uncertainties associated with the association between climate-induced changes in meteorological conditions and the incidence of wildland fire events.^50^ The existing literature, including our study, accounts partially for the role of meteorological conditions but not for the influence of climate-driven changes in wildland fires. Using a multimodel ensemble, Park and colleagues^51^ found that the RCP8.5 scenario yielded substantial global excess of deaths associated with PM_2.5_, including North America, compared with a year 2000 baseline.

This study adds to the literature by examining 2 climate models at 4 future time periods and simulating the influence of reduced air pollutant emissions. We found that reducing anthropogenic sources of PM_2.5_ and O_3_ precursor emissions would substantially attenuate, but not fully mitigate, the association of future changes in climate-driven air quality with human health outcomes.

## Conclusions

This modeling study estimated the number of premature deaths associated with PM_2.5_ and O_3_ using concentration-response parameters drawn from epidemiological studies. This literature characterizes the risks associated with historical changes in air pollution levels. To the extent that these risks are sensitive to meteorologically influenced behavioral variables (such as air conditioning use), then using these parameters to estimate future changes in risk may underestimate or overestimate impacts. The magnitude of these uncertainties is unknown. These uncertainties notwithstanding, this study adds to a larger body of literature examining the role of climate change in future air pollutant levels by simulating the role of reduced anthropogenic emissions in mitigating these effects.

## Notes

Supplement. **eAppendix.** Representative Concentration Pathways **eTable 1.** Domain-Wide Annual Total Anthropogenic Emissions Used in Air Quality Modeling **eTable 2.** Epidemiological Study Parameters Used to Quantify Risks Associated With PM_2.5_ **eTable 3.** All-Cause Mortality Rate by Source, Year, and Age Group **eTable 4.** Ratio of Future Year All-Cause Mortality Rate to 2013 Estimated All-Cause Mortality Rate by Age Group **eTable 5.** Projected Annual Mean National Temperature Change by Model and Year **eTable 6.** Projected Population-Weighted PM_2.5_ and Ozone Concentrations Associated With Future Changes in Climate: 2025, 2050, 2075, and 2095 **eTable 7.** Number of Excess or Avoided PM_2.5_- and Ozone-Attributable Premature Deaths by Region: 2025, 2050, 2075, and 2095 **eFigure 1.** Biases in Model-Projected Temperatures (1995-2005) **eFigure 2.** Model-Projected Changes in Summer Season Temperature (2030 to 2095) **eFigure 3.** Model-Projected Changes in May to September Maximum Daily 8-Hour Mean Ozone Concentrations (2030 to 2095) **eFigure 4.** Model-Projected Changes in Annual Mean PM_2.5_ Concentrations (2030 to 2095) **eFigure 5.** Sum of PM_2.5_- and Ozone-Attributable Premature Deaths by State Estimated Using the CESM Model and 2011 and 2040 Emission Inventories: 2030, 2050, 2075, and 2095 **eFigure 6.** Sum of PM_2.5_- and Ozone-Attributable Premature Deaths by State Estimated Using the CM3 Model and 2011 and 2040 Emission Inventories: 2030, 2050, 2075, and 2095 **eReferences**
