# Supplementary material for: Associations Between Simulated Future Changes in Climate, Air Quality, and Human Health
Source: JAMA Netw Open. 2021 Jan 4;4(1):e2032064. doi: 10.1001/jamanetworkopen.2020.32064 (PMC7783541; doi:10.1001/jamanetworkopen.2020.32064)
Supplement: Supplement. — eAppendix. Representative Concentration Pathways eTable 1. Domain-Wide Annual Total Anthropogenic Emissions Used in Air Quality Modeling eTable 2. Epidemiological Study Parameters Used to Quantify Risks Associated With PM2.5 eTable 3. All-Cause Mortality Rate by Source, Year, and Age Group eTable 4. Ratio of Future Year All-Cause Mortality Rate to 2013 Estimated All-Cause Mortality Rate by Age Group eTable 5. Projected Annual Mean National Temperature Change by Model and Year eTable 6. Projected Population-Weighted PM2.5 and Ozone Concentrations Associated With Future Changes in Climate: 2025, 2050, 2075, and 2095 eTable 7. Number of Excess or Avoided PM2.5- and Ozone-Attributable Premature Deaths by Region: 2025, 2050, 2075, and 2095 eFigure 1. Biases in Model-Projected Temperatures (1995-2005) eFigure 2. Model-Projected Changes in Summer Season Temperature (2030 to 2095) eFigure 3. Model-Projected Changes in May to September Maximum Daily 8-Hour Mean Ozone Concentrations (2030 to 2095) eFigure 4. Model-Projected Changes in Annual Mean PM2.5 Concentrations (2030 to 2095) eFigure 5. Sum of PM2.5- and Ozone-Attributable Premature Deaths by State Estimated Using the CESM Model and 2011 and 2040 Emission Inventories: 2030, 2050, 2075, and 2095 eFigure 6. Sum of PM2.5- and Ozone-Attributable Premature Deaths by State Estimated Using the CM3 Model and 2011 and 2040 Emission Inventories: 2030, 2050, 2075, and 2095 eReferences [file jamanetwopen-e2032064-s001.pdf]

# Supplemental Online Content

Fann NL, Nolte CG, Sarofim MC, Martinich J, Nassikas NJ. Associations between simulated future changes in climate, air quality, and human health. *JAMA Netw Open*. 2021;4(1):e2032064. doi:10.1001/jamanetworkopen.2020.32064

**eAppendix.** Representative Concentration Pathways
**eTable 1.** Domain-Wide Annual Total Anthropogenic Emissions Used in Air Quality Modeling
**eTable 2.** Epidemiological Study Parameters Used to Quantify Risks Associated With $PM_{2.5}$
**eTable 3.** All-Cause Mortality Rate by Source, Year, and Age Group
**eTable 4.** Ratio of Future Year All-Cause Mortality Rate to 2013 Estimated All-Cause Mortality Rate by Age Group
**eTable 5.** Projected Annual Mean National Temperature Change by Model and Year
**eTable 6.** Projected Population-Weighted $PM_{2.5}$ and Ozone Concentrations Associated With Future Changes in Climate: 2025, 2050, 2075, and 2095
**eTable 7.** Number of Excess or Avoided $PM_{2.5}$- and Ozone-Attributable Premature Deaths by Region: 2025, 2050, 2075, and 2095
**eFigure 1.** Biases in Model-Projected Temperatures (1995-2005)
**eFigure 2.** Model-Projected Changes in Summer Season Temperature (2030 to 2095)
**eFigure 3.** Model-Projected Changes in May to September Maximum Daily 8-Hour Mean Ozone Concentrations (2030 to 2095)
**eFigure 4.** Model-Projected Changes in Annual Mean $PM_{2.5}$ Concentrations (2030 to 2095)
**eFigure 5.** Sum of $PM_{2.5}$- and Ozone-Attributable Premature Deaths by State Estimated Using the CESM Model and 2011 and 2040 Emission Inventories: 2030, 2050, 2075, and 2095
**eFigure 6.** Sum of $PM_{2.5}$- and Ozone-Attributable Premature Deaths by State Estimated Using the CM3 Model and 2011 and 2040 Emission Inventories: 2030, 2050, 2075, and 2095
**eReferences**

This supplemental material has been provided by the authors to give readers additional information about their work.

© 2021 Fann N et al. *JAMA Network Open.*

**eAppendix.** Representative Concentration Pathways

The Earth's global mean temperature is determined by the balance between incoming solar radiation and terrestrial energy radiated back to space. Atmospheric greenhouse gases absorb infrared radiation, causing the temperature to increase to maintain equilibrium. Because the time scale for the Earth's climate to reach equilibrium is long, anthropogenic warming is often measured by the difference in radiant energy, or *radiative forcing*, relative to preindustrial conditions in 1750.

Future greenhouse gas concentration trajectories known as Representative Concentration Pathways (RCPs) have been developed for modeling climate change and resulting impacts. These trajectories are named according to their year 2100 radiative forcing, measured in Watts per square meter. Due to computational constraints, the only scenario used in this paper is RCP8.5. RCP8.5 is a pathway with relatively high greenhouse gas concentrations, leading to substantial warming by 2100.. RCP8.5 is selected to assess a wide range of future temperatures, but this does not imply a judgment regarding the likelihood of that scenario.

© 2021 Fann N et al. *JAMA Network Open.*

**eTable 1.** Domain-Wide Annual Total Anthropogenic Emissions Used in Air Quality Modeling[I]

| **Species** | **2011**[B] | **2040** [C] | **Relative Change** |
|---|---|---|---|
| | | | |
| Nitrogen Oxides | 16,993,249 | 9,593,297 | -43.5% |
| Sulfur Dioxide | 7,730,734 | 3,291,595 | -57.4% |
| Volatile Organic Carbons | 19,379,305 | 17,004,702 | -12.3% |
| $PM_{2.5}$ | 5,007,957 | 4,990,098 | -0.4% |

[A] Biogenic (i.e., vegetative) emissions of volatile organic compounds (VOC), including isoprene and other terpenes, were estimated by the model using the downscaled meteorology.
[B] Taken from Table 5-1 of U.S. EPA. *Emissions Inventory for Air Quality Modeling Technical Support Document: Heavy-Duty Vehicle Greenhouse Gas Phase 2 Final Rule*, EPA-420-R-16-008, Washington, DC; 2016.
[C] Taken from Table 5-2 of U.S. EPA. *Emissions Inventory for Air Quality Modeling Technical Support Document: Heavy-Duty Vehicle Greenhouse Gas Phase 2 Final Rule*, EPA-420-R-16-008, Washington, DC; 2016.
Abbreviations: $PM_{2.5}$, particulate matter smaller than 2.5 microns in diameter.

© 2021 Fann N et al. *JAMA Network Open.*

**eTable 2.** Epidemiological Study Parameters Used to Quantify Risks Associated With $PM_{2.5}$

| Endpoint | Study | Study Population | Risk Estimate (95P PPercent Confidence Interval)P |
|---|---|---|---|
| **Premature Mortality** | | | |
| Cohort study, all-cause | Krewski et al. (2009)[2] | >29 years | HR = 1.06 (1.04 – 1.08) per 10 µg/mP[3] |
| Time Series, non-accidental | Zanobetti & Schwartz (2008)[3] | All Ages | Percent Increase = 0.53% (95% CI: 0.28-0.77) per 10ppb |

[A] Beta coefficients derived from Hazard Ratio and percent change in risk

© 2021 Fann N et al. *JAMA Network Open.*

**Procedure for projecting death rates to future years**

The BenMAP-CE program contains age- and cause-stratified death rates for each county in the contiguous U.S. through the year 2060 in 5-year increments. To estimate these rates, we calculated annual adjustment factors, based on a series of Census Bureau projected national mortality rates (for all- cause mortality), to adjust the age- and county-specific mortality rates calculated using an average of 2012-2014 data from the CDC-WONDER database as a baseline (eTable 3), projected using ratios reported by the U.S. Census Bureau (eTable 4). We used the following procedure:

1. For each age group, we obtained the series of projected national mortality rates from 2013 to 2050 (see the 2013 rate in eTable 3 below) based on Census Bureau projected life tables.

2. We then calculated, separately for each age group, the ratio of Census Bureau national mortality rate in year Y (Y = 2014, 2015, ..., 2060) to the 2013 rate. These ratios are shown for selected years in eTable 4.

3. Finally, to estimate mortality rates in year Y (Y = 2015, 2020, ..., 2060) that are both age-group-specific and county-specific, we multiplied the county- and age-group-specific mortality rates for 2012-2014 by the appropriate ratio calculated in the previous step. For example, to estimate the projected mortality rate in 2030 among ages 18-24 in Wayne County, MI, we multiplied the mortality rate for ages 18-24 in Wayne County in 2012-2014 by the ratio of Census Bureau projected national mortality rate in 2030 for ages 18-24 to Census Bureau national mortality rate in 2013 for ages 18-24.

**eTable 3.** All-Cause Mortality Rate by Source, Year, and Age Group

| ***Source & Year*** | ***18-24*** | ***25-34*** | ***35-44*** | ***45-54*** | ***55-64*** | ***65-74*** | ***75-84*** | ***85+*** |
|---|---|---|---|---|---|---|---|---|
| *Calculated CDC 2012-2014* | 0.078 | 0.107 | 0.173 | 0.405 | 0.862 | 1.797 | 4.628 | 13.580 |
| *Census Bureau 2013*[A] | 0.088 | 0.102 | 0.183 | 0.387 | 0.930 | 2.292 | 5.409 | 13.091 |

[A] For a detailed description of the model, the assumptions, and the data used to create Census Bureau projections, see the working paper, “Methodology and Assumptions for the 2012 National Projections,” which is available on http://www.census.gov/population/projections/files/methodology/methodstatement12.pdf

© 2021 Fann N et al. *JAMA Network Open.*

**eTable 4.** Ratio of Future Year All-Cause Mortality Rate to 2013 Estimated All-Cause Mortality Rate by Age Group

| **Year** | **Infant** | **1-17** | **18-24** | **25-34** | **35-44** | **45-54** | **55-64** | **65-74** | **75-84** | **85+** |
|---|---|---|---|---|---|---|---|---|---|---|
| 2030 | 0.81 | 0.75 | 0.66 | 0.70 | 0.67 | 0.69 | 0.78 | 0.86 | 0.89 | 0.92 |
| 2050 | 0.67 | 0.56 | 0.39 | 0.40 | 0.40 | 0.44 | 0.53 | 0.66 | 0.77 | 0.87 |
| 2060[A] | 0.61 | 0.48 | 0.30 | 0.30 | 0.31 | 0.34 | 0.43 | 0.58 | 0.70 | 0.87 |

[A] Used when quantifying air pollution-attributable deaths for the years 2075 and 2095

© 2021 Fann N et al. *JAMA Network Open.*

**eTable 5.** Projected Annual Mean National Temperature Change by Model and Year

| | ***CM3*** | ***CESM*** |
|---|---|---|
| *2030* | 2.0 | 1.5 |
| *2050* | 3.1 | 2.3 |
| *2075* | 5.2 | 3.5 |
| *2095* | 6.6 | 4.7 |

© 2021 Fann N et al. *JAMA Network Open.*

**eTable 6.** Projected Population-Weighted $PM_{2.5}$ and Ozone Concentrations Associated With Future Changes in Climate: 2025, 2050, 2075, and 2095

| | **CM3** | | | | **CESM** | | | |
|---|---|---|---|---|---|---|---|---|
| | *2011* | | *2040* | | *2011* | | *2040* | |
| | PM | Ozone | PM | Ozone | PM | Ozone | PM | Ozone |
| **2030** | 0.14 | 0.82 | 0.07 | -0.08 | 0.08 | 1.6 | 0.07 | 0.94 |
| **2050** | 0.18 | 1.08 | 0.10 | -0.35 | 0.08 | 1.4 | 0.10 | 0.70 |
| **2075** | 0.57 | 2.70 | 0.38 | 0.34 | 0.11 | 2.3 | 0.10 | 0.93 |
| **2095** | 0.65 | 3.56 | 0.46 | 0.59 | 0.14 | 3.3 | 0.13 | 1.4 |

© 2021 Fann N et al. *JAMA Network Open.*

**eTable 7.** Number of Excess or Avoided $PM_{2.5}$- and Ozone-Attributable Premature Deaths by Region: 2025, 2050, 2075, and 2095

| | ***CM3*** | | | | ***CESM*** | | | |
|---|---|---|---|---|---|---|---|---|
| | **2011** | | **2040** | | **2011** | | **2040** | |
| **Region** | *PM* | *Ozone* | *PM* | *Ozone* | *PM* | *Ozone* | *PM* | *Ozone* |
| *Northwest* | 820 | 120 | 770 | 28 | 260 | 100 | 270 | 48 |
| *Southwest* | 5,300 | 360 | 4,800 | -170 | 1,900 | 340 | 2,100 | 60 |
| *Northern Great Plains* | -90 | 54 | -18 | 27 | -73 | 57 | -24 | 43 |
| *Southern Great Plains* | 2,300 | 320 | 2,500 | 40 | -690 | 110 | 48 | -12 |
| *Southeast* | 11,000 | 720 | 7,100 | -270 | 4,800 | 580 | 3,700 | -31 |
| *Midwest* | 1,200 | 1,300 | -1,100 | 670 | -2,800 | 1,400 | -1,800 | 960 |
| *Northeast* | 3,500 | 1,300 | 1,100 | 320 | 1,400 | 1,200 | 260 | 550 |

© 2021 Fann N et al. *JAMA Network Open.*

**eFigure 1.** Biases in Model-Projected Temperatures (1995-2005)[4]

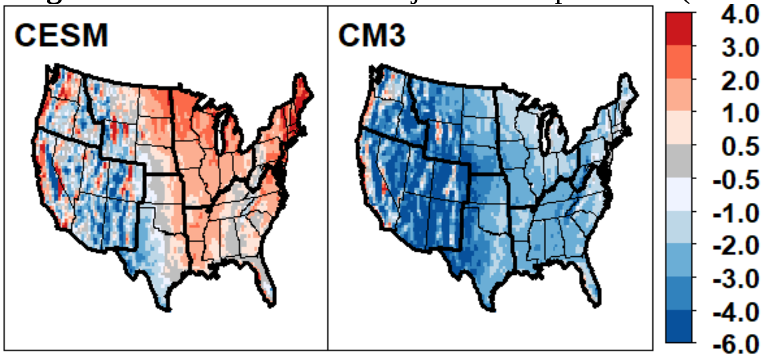


© 2021 Fann N et al. *JAMA Network Open.*

**eFigure 2.** Model-Projected Changes in Summer Season Temperature (2030 to 2095)

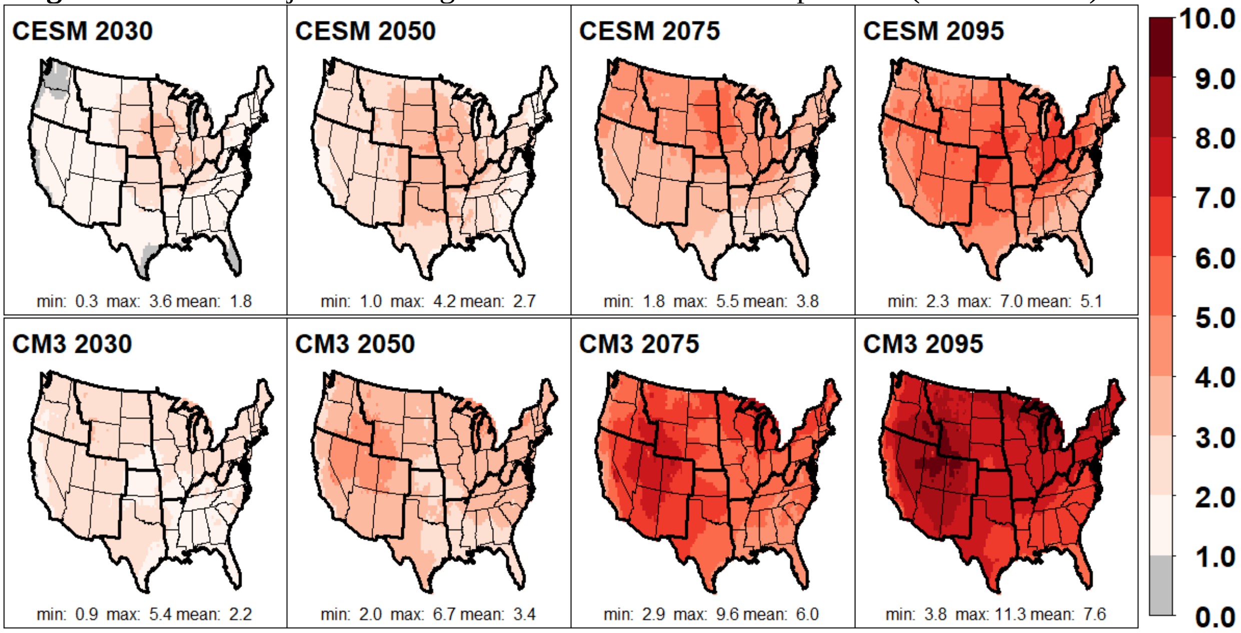


© 2021 Fann N et al. *JAMA Network Open.*

**eFigure 3.** Model-Projected Changes in May to September Maximum Daily 8-Hour Mean Ozone Concentrations (2030 to 2095)

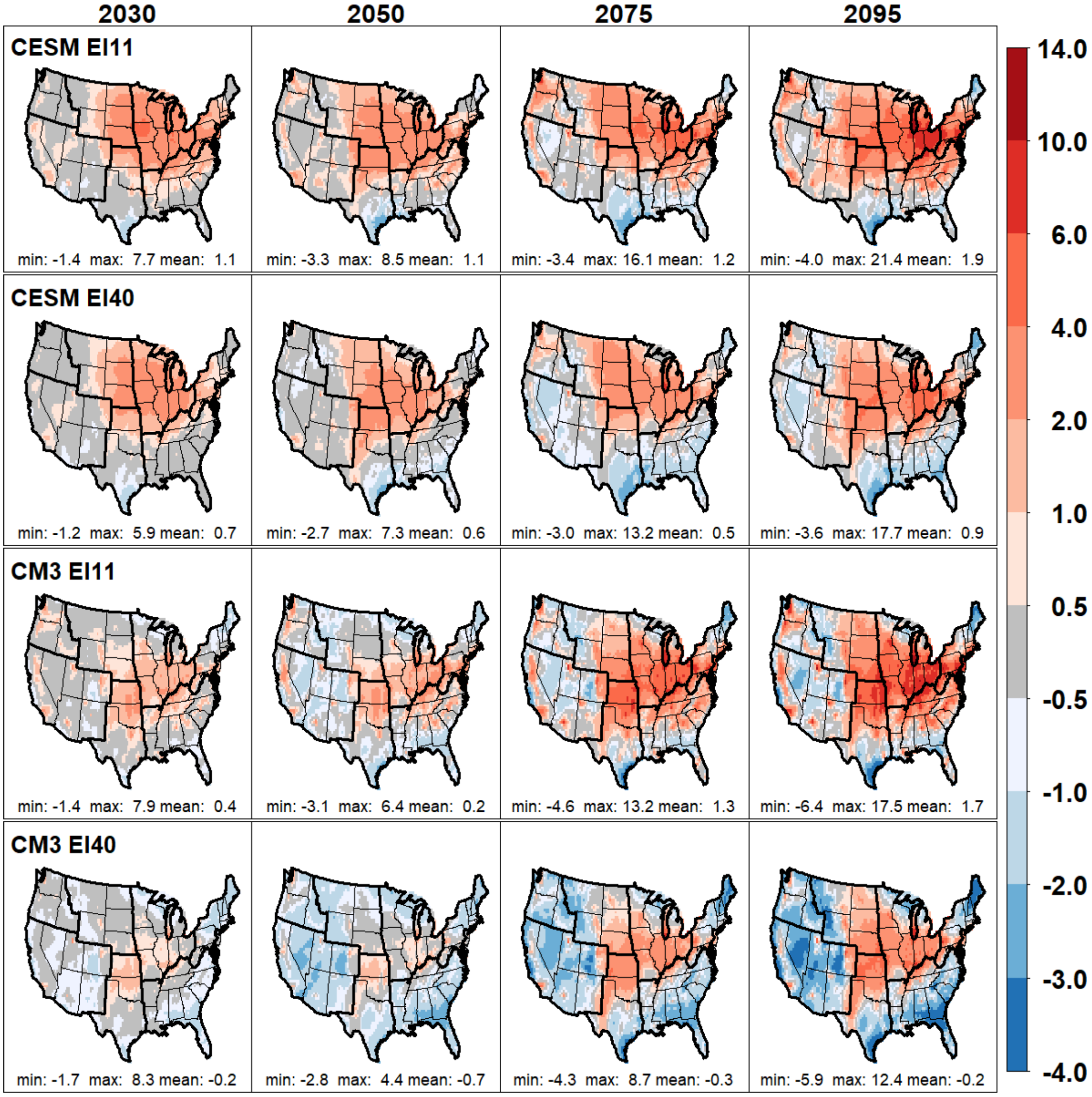


© 2021 Fann N et al. *JAMA Network Open.*

**eFigure 4.** Model-Projected Changes in Annual Mean $PM_{2.5}$ Concentrations (2030 to 2095)

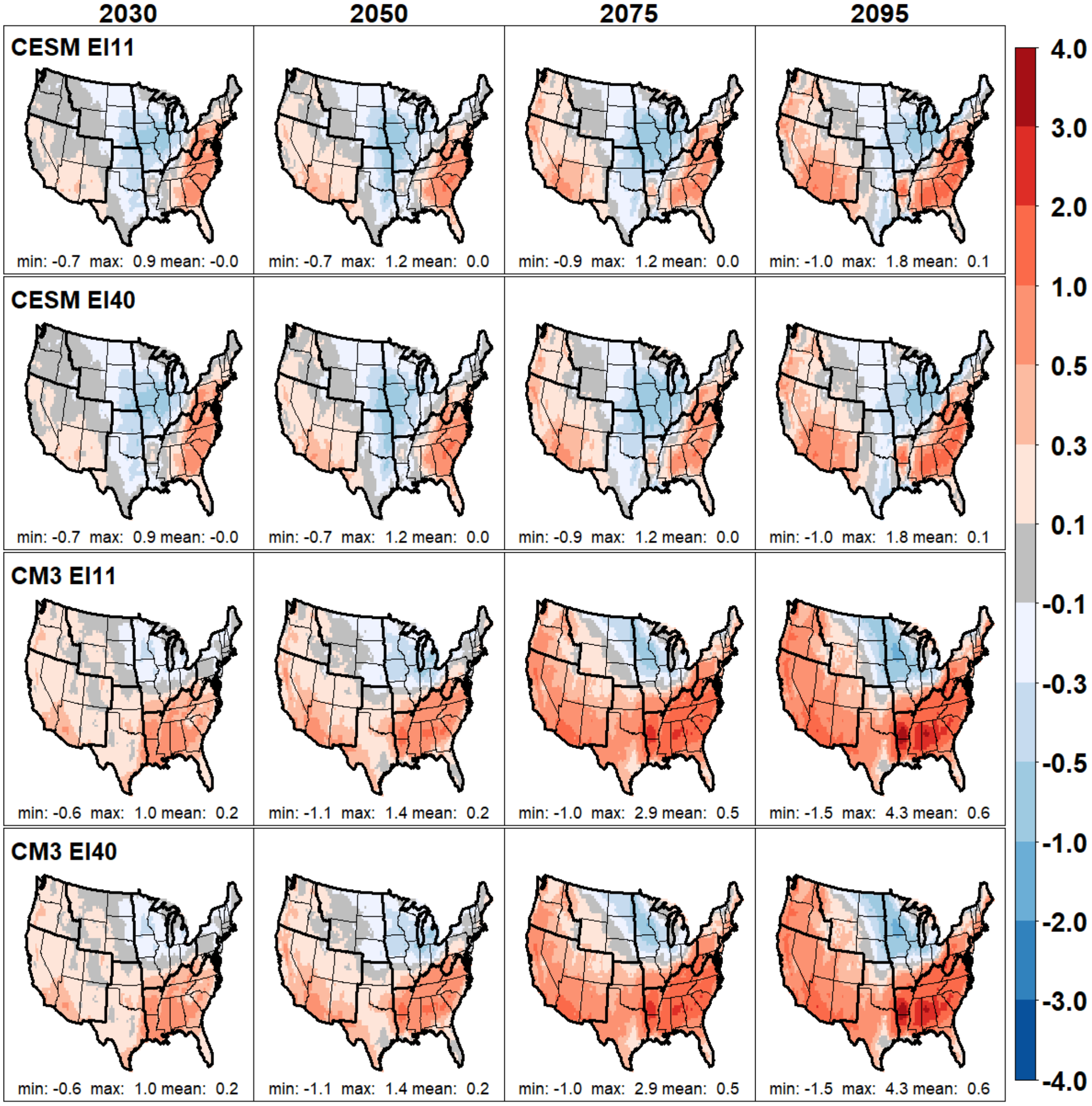


© 2021 Fann N et al. *JAMA Network Open.*

**eFigure 5.** Sum of $PM_{2.5}$- and Ozone-Attributable Premature Deaths by State Estimated Using the CESM Model and 2011 and 2040 Emission Inventories: 2030, 2050, 2075, and 2095

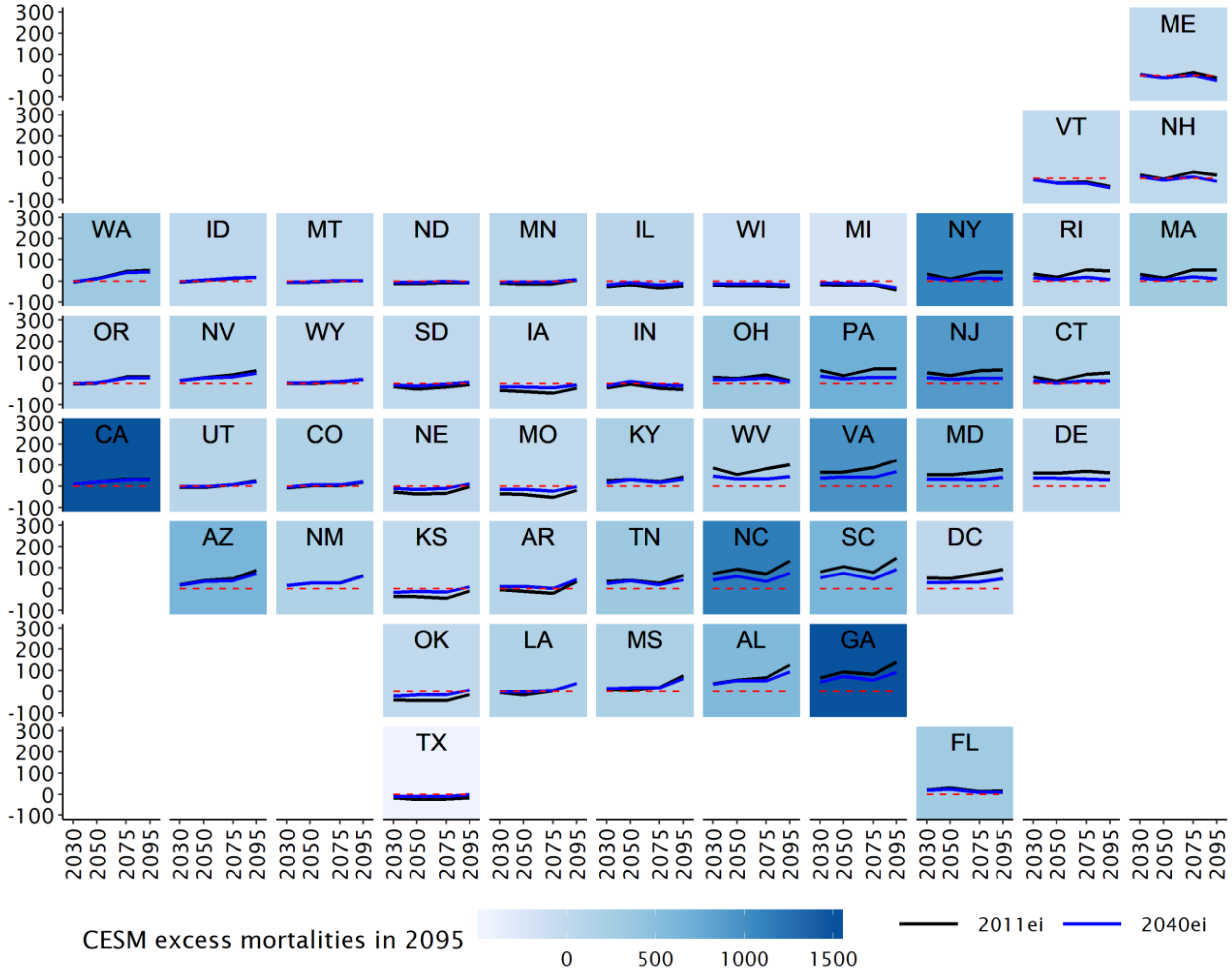


© 2021 Fann N et al. *JAMA Network Open.*

**eFigure 6.** Sum of $PM_{2.5}$- and Ozone-Attributable Premature Deaths by State Estimated Using the CM3 Model and 2011 and 2040 Emission Inventories: 2030, 2050, 2075, and 2095

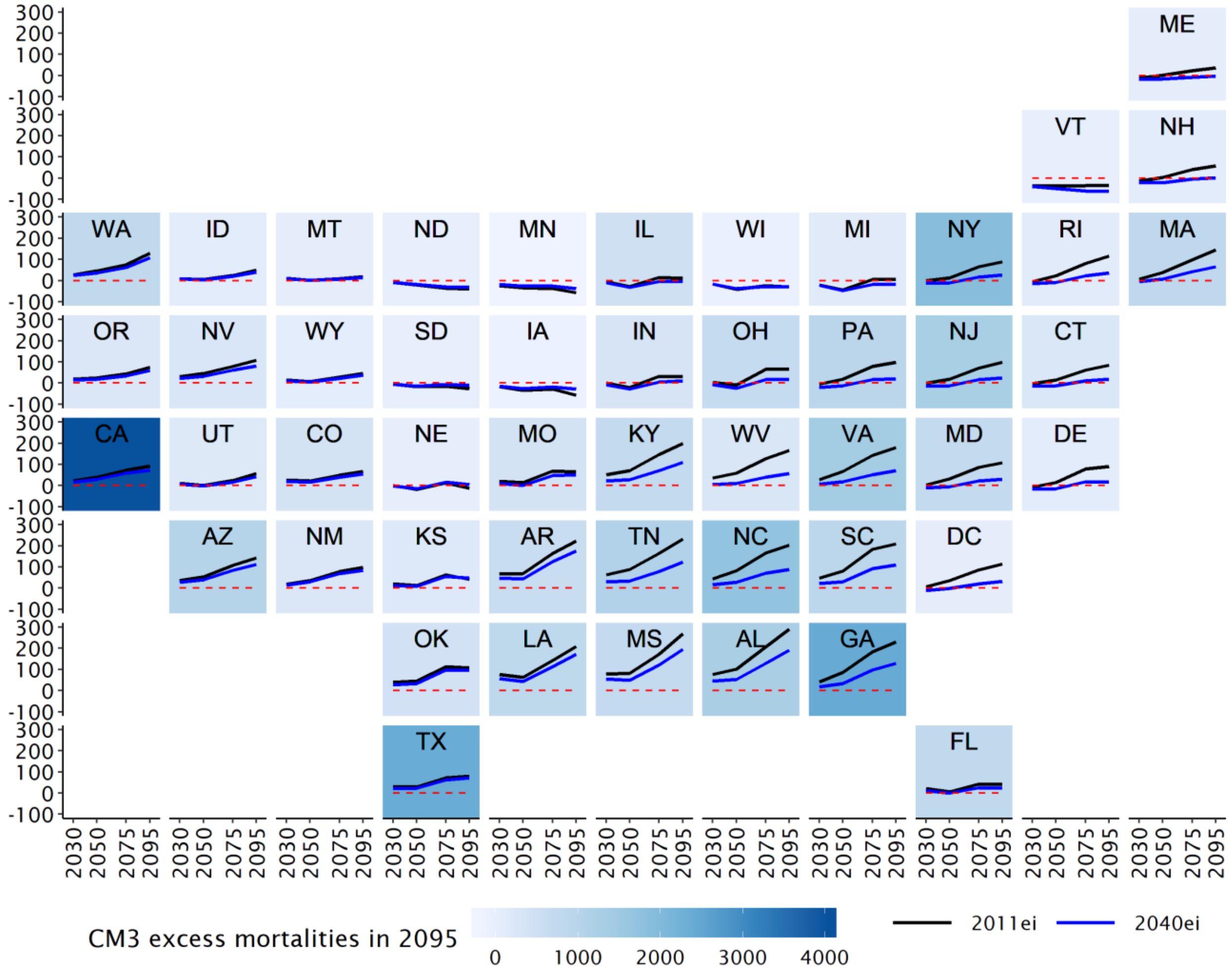


© 2021 Fann N et al. *JAMA Network Open.*

## eReferences

1. U.S. EPA. *Emissions Inventory for Air Quality Modeling Technical Support Document: Heavy-Duty Vehicle Greenhouse Gas Phase 2 Final Rule*; Washington, DC, 2016. https://doi.org/EPA-420-R-16-008.
2. Krewski, D.; Jerrett, M.; Burnett, R. T.; Ma, R.; Hughes, E.; Shi, Y.; Turner, M. C.; Pope, C. A.; Thurston, G.; Calle, E. E.; et al. Extended Follow-up and Spatial Analysis of the American Cancer Society Study Linking Particulate Air Pollution and Mortality. *Res. Rep. Health. Eff. Inst.* **2009**, No. 140, 5–114; discussion 115-36.
3. Zanobetti, A.; Schwartz, J. Is There Adaptation in the Ozone Mortality Relationship: A Multi-City Case-Crossover Analysis. *Environ. Health* **2008**, *7*, 22. https://doi.org/10.1186/1476-069X-7-22.
4. Saha, S.; Moorthi, S.; Pan, H. L.; Wu, X.; Wang, J.; Nadiga, S.; Tripp, P.; Kistler, R.; Woollen, J.; Behringer, D.; et al. The NCEP Climate Forecast System Reanalysis. *Bull. Am. Meteorol. Soc.* **2010**, *91* (8), 1015–1057. https://doi.org/10.1175/2010BAMS3001.1.

© 2021 Fann N et al. *JAMA Network Open.*
